# Feasibility and acceptability of a multi-level school-based intervention for adolescent mental health in Nepal: a mixed-methods study

**DOI:** 10.1186/s13034-026-01111-7

**Published:** 2026-06-13

**Authors:** Nagendra Prasad Luitel, Indira Pradhan, Bishnu Lamichhane, Nishan Dulal, G. J. Melendez-Torres, Emily Shi, Malka Dhillon, Kamal Gautam, Sarah Skeen, Mark Tomlinson, Mark J. D. Jordans

**Affiliations:** 1 Department of Global Public Health, Karolinska Institutet, Stockholm, Sweden; 2 Research Department, Transcultural Psychosocial Organization Nepal (TPO Nepal), Baluwatar, Kathmandu, Nepal; 3 Center for Global Mental Health Equity, Department of Psychiatry and Behavioural Health, The George Washington University, Washington, DC USA; 4 University of Exeter, Exeter, UK; 5 University of Illinois Chicago College of Medicine, Peoria, IL USA; 6 Columbia University, Columbia, NY USA; 7 Department of Global Health, Institute for Life Course Health Research, Stellenbosch University, Cape Town, South Africa; 8 Mental Health, Alcohol, Substance useand Tobacco Research Unit, South African Medical Research Council, Cape Town, South Africa; 9 School of Nursing and Midwifery, Queens University, Belfast, UK; 10 Health Service and Population Research Department, Centre for Global Mental Health, Institute of Psychiatry, Psychology and Neuroscience, King’s College London, London, UK

**Keywords:** Mental health promotion, School-based, School climate, Nepal, Depression and anxiety

## Abstract

**Background:**

Adolescence is a crucial developmental stage characterized by rapid biological, psychological, and social changes, that increase vulnerability to mental health issues. Nearly half of all mental health conditions have their onset during this period and are influenced by individual, family, and environmental factors. Schools play a key role in promoting mental well-being among adolescents due to their accessibility and reach. While school-based mental health prevention interventions have shown positive outcomes in high-income countries, evidence from low- and middle-income countries (LMICs) is limited. This study aimed to assess the feasibility and acceptability of Health Action in Schools for a Thriving Adolescent Generation (HASHTAG), a comprehensive school-based intervention for adolescents aged 13–16 years in Nepal.

**Methods:**

A feasibility cluster-randomized controlled trial was conducted in four secondary schools in Morang district, Nepal. Adolescents completed surveys at baseline and at a 3-month follow-up to evaluate mental well-being, emotional and behavioral outcomes, social support, school climate, and functioning. The intervention comprised two components: Thriving Environment in Schools (TES), implemented over three months and Thriving Together (TT), delivered through six weekly sessions. Quantitative data were collected electronically and analyzed descriptively. Qualitative data were gathered through focus group discussions and in-depth interviews with adolescents, teachers, and facilitators to explore implementation experiences and were analyzed thematically.

**Results:**

Both the TES and TT components were well received, with TT sessions achieving an average attendance rate exceeding 70%, and being delivered as planned by trained facilitators, indicating good acceptability and feasibility. Explanatory analysis suggested a positive trend in anxiety outcomes in the intervention group while, social support showed a modest increase. Other outcomes showed small, non-significant changes. Qualitative findings highlighted perceived benefits such as improvements in school cleanliness, staff–student relationships, bullying and discrimination reduction, and positive social and behavioral changes. Participants found breathing exercises, games, the workbook, and the ‘feeling box’ particularly helpful. Implementation challenges included limited space, logistical constraints, COVID-19 related disruptions, and session length.

**Conclusion:**

HASHTAG demonstrated feasibility and accessibility as a school-based intervention for adolescents in Nepal, with preliminary indications of potential benefits. Addressing identified implementation challenges will be important for optimizing delivery in future studies. A fully powered randomized controlled trial is warranted to evaluate effectiveness.

## Introduction

 Adolescence is a crucial phase characterized by significant physical, emotional, and social changes. Around 90% of adolescents globally live in low- to middle-income countries (LMIC) [1]. These countries often face structural vulnerabilities and limited resources [2], leading to factors like poverty, abuse, and violence that can increase the risk of mental health issues such as depression, anxiety, and suicidal thoughts [3]. This heightened exposure to risk factors put millions of adolescents in LMICs at a greater risk for poor mental health outcomes. Mental disorders typically emerge during adolescence and are worsened by known risk factors, with almost half of all lifetime conditions starting by mid-adolescence [4]. Depression and anxiety are prevalent among young people, accounting for a significant portion (25%) of years lived with disability and contributing to excess mortality from suicide [5]. Various factors, including individual traits, family dynamics, and external influences, play a role in adolescent mental health [6–9]. Addressing these factors is crucial for promoting overall well-being among adolescents.

Nepalese adolescents face a unique combination of mental health vulnerabilities and limited access to dedicated services [10]. They have experienced recent and long-standing traumatic events, such as major earthquakes in 2015, the Maoist insurgency from 1996 to 2006, and recurring natural disasters like annual floods and landslides, all within a context of socio-economic challenges. Research on child and adolescent mental health in Nepal is limited, with studies reporting varying prevalence rates of depression ranging from 0.4% to 56.5% [11–18] and anxiety between 2% and 55.6% [11, 14, 17–20] among children and adolescents. Despite the significant mental health risks faced by Nepalese adolescents, there is a lack of evidence on effective, wide-spread interventions.

While school-based mental health promotion programs have shown positive outcomes in high-income countries, research in LMICs like Nepal is limited [21, 22]. Systemic barriers, including insufficient mental health infrastructure, a shortage of trained professionals, and stigma, impedes mental health interventions in Nepal [23–25]. Although there have been some studies on school-based mental health interventions in Nepal [26, 27], they have mainly focused on treatment rather than prevention strategies [28]. It is essential to comprehend how Nepalese adolescents view school-based mental health promotion, and the influence of cultural adaptation on the effectiveness of interventions [29].

Preventive interventions can help reduce the severity of mental health issues and prove to be cost-effective in the long term, potentially decreasing the need for extensive treatment and associated costs [30, 31]. Schools are an ideal setting for promoting adolescent mental health due to their accessibility and resources. Additionally, school and community-based approaches have been successful in improving depression, social functioning, academic performance, and health behaviours [32]. The National Adolescent Health and Development Strategy of Nepal emphasizes the importance of school-based psychosocial activities for mental health promotion [33]; however, no large-scale school-based intervention has been developed and implemented. To address this gap, the Health Action in ScHools for a Thriving Adolescent Generation (HASHTAG) intervention was developed for adolescents aged 13–16 years, focusing on mental-well-being and coping skills [34]. This feasibility trial examines the implementation process, challenges, and facilitators of the program, evaluating its feasibility, acceptability, and appropriateness from the perspectives of students, teachers, and facilitators. The findings could guide government initiatives to integrate mental health promotion in schools [33], and align with global recommendations supporting school-based health initiatives to enhance student well-being [35]. This study contributes to the growing body of research supporting the importance, feasibility, and potential benefits of universal mental health interventions in school settings.

## Methods

### Study design

The study used a feasibility cluster randomized controlled trial (c-RCT) to assess the feasibility, acceptability and trial process, with schools as the unit of randomization [36]. Data on implementation, including session attendance, dropout rates, reasons for dropout, and challenges, were collected throughout the implementation period. A qualitative study combining Focus Group Discussions (FGDs), and Individual Interviews (IDIs), was conducted post-intervention with students, teachers and facilitators to evaluate the feasibility, acceptability, and impact of the intervention.

### Study area

The study was conducted in Morang, an eastern district of Nepal consisting of 17 municipalities or rural municipalities. The district has a total population of 1,148,156 according to the 2021 census, is known for its diversity in terms of caste/ethnicity, language, and geography. Kanepokhari rural municipality was selected purposively due to its diverse population, the lack of school-based mental health interventions, high reported prevalence of suicide, and local government support. Kanepokhari has a population of 43,193 with 10,663 households and a literacy rate of 77.8%, slightly higher than the national average of 76.2% [37].

### Overview of the intervention

The HASHTAG intervention consists of two modules: Thriving Environment in Schools (TES), implemented over three months and Thriving Together (TT) delivered through six weekly sessions. These modules were developed collaboratively with adolescents, parents, teachers, school leaders, community members, and education officials in South Africa and Nepal. Originally developed in English, the intervention was translated and culturally adapted for Nepal following recommended adaptation procedures [38, 39]. TES aims to enhance the school climate by strengthening connections and supportive relationships through a comprehensive school-wide approach. It involves the establishment of School Action Groups (SAGs) to coordinate school-wide activities, a two-day teacher training on stress management and self-care, and student mental health awareness campaigns conducted through school cleanliness and writing competitions. TT is a six-session group program for young adolescents delivered by trained facilitators. Drawing on evidence from systematic reviews [40], it incorporates seven proven components—interpersonal skills, emotional regulation, stress management, mindfulness, problem-solving, assertiveness, and alcohol/drug education, which are reinforced through repeated practice across sessions [34].

The Government of Nepal has recently introduced the “One School, One Nurse” program in secondary schools to improve health outcomes for students. This initiative involves deploying certified nurses in community schools to provide preventive healthcare, health education, and first-aid services [41]. To support this policy, staff nurses received training to ensure program’s sustainability beyond the HASHTAG project. Four staff nurses underwent a 15-day training program divided into two phases. The first phase focused on Foundational Helping Skills (FHS) [42] and group management skills emphasizing effective communication, understanding psychosocial needs, assessing risk behaviours, and managing group activities. The second phase included training on stress management, problem solving, emotional regulation, and assertiveness skills, using interactive methods such as presentations, role plays, and group activities. A psychosocial counsellor was available to provide immediate support to individuals reporting suicidal thoughts or self-harm during baseline and follow-up interviews, as well as during the implementation of the HASHTAG intervention. Research assistants and facilitators were trained to identify and refer adolescents at risk of suicide. An Adverse Event Reporting and Management (AERM) protocol [43], previously used in our studies, was implemented to assess and manage adverse events, including suicidal thoughts or self-harm.

### Participants

The pilot trial involved adolescents between the age of 13 and 16, and the qualitative component included adolescents, teachers, and facilitators who were involved in the implementation of the TT and TES programs. Eligible adolescent participants were students in grades 8 and 9, aged 13 and 16, at the chosen schools. They were required to be fluent in Nepali, provide assent, have parental or caregiver consent, and have completed the baseline survey.

### Sample size and sampling

This feasibility trial was conducted in four schools to evaluate the feasibility and acceptability of the HASHTAG intervention, as well as the trial process for a potential future fully powered RCT. The sample size for this pilot trial was determined pragmatically rather than through formal power calculations for effectiveness outcomes. The target sample size approximately 60 participants per school was set to assess recruitment, retention, data collection procedures, and intervention delivery at the school level, resulting in a total intended sample size of 120 adolescents per trial arm and 240 participants overall. All eligible students in Grades 8 and 9 in the selected schools were invited to participate, with the final number of participants varying across schools and trial arms due to differences in school size and student enrolment. In one control school with a smaller student population in Grades 8 and 9, 53 participants were recruited instead of the target sample size. Adolescents in the control schools were provided with information about available mental health services in the district.

FGDs and IDIs were conducted with students, teachers, and facilitators to collect their insights and opinions on the HASHTAG intervention. Two FGDs (*N* = 20) were conducted with students who took part in the TT sessions, two FGDs (*N* = 12) with SAGs members (students), and one FGD with facilitators (*N* = 4). Additionally, ten IDIs were held with teachers who were either SAGs members or involved in implementing the intervention.

### Clinical and psychological outcome measures

Clinical and psychological outcomes are the secondary measures in this feasibility study that assess the sensitivity of key constructs to change before-and-after an intervention. These measures are important for determining the sample size needed for a subsequent randomized controlled trial (RCT). The main secondary outcomes of this feasibility trial included mental well-being, symptoms of depression and anxiety in the post-intervention assessment. Mental well-being was assessed using the 15-item Stirling Children’s Wellbeing Scale [44]. Depression and anxiety symptoms were evaluated using the Patient Health Questionnaire 9-items modified for adolescents (PHQ-A) [45] and the General Anxiety Disorder 7-item (GAD-7) respectively [46]. Both the PHQ-A and GAD-7 have been validated in Nepal [47]. Social behaviours were evaluated using a 5-item sub-scale from the Strength and Difficulties Questionnaire (SDQ) [48] which has been previously used in Nepal [49]. Substance use was measured using the Alcohol Use Disorders Identification Test [50] which has been validated and widely used in Nepal [51, 52]. Adolescents’ difficulties in performing day-to-day activities were evaluated using the World Health Organization Disability Assessment Schedule (WHODAS 2.0) Children and Youth Version [53]. We used only the Life Activities domain of the 36-items instrument, which comprises four items related to household activities and five items related to school activities. Aggression among students was measured using an 11-item self-reported aggressive behaviors scale for young adolescents [54]. Resilience among adolescents was assessed using a 9-item Resilience Scale adapted from the 26-Item [55] which has been used in the Child Soldiers Study in Nepal [56]. Experience of bullying in school was evaluated using the 12-item Gatehouse Bullying Scale [57], and school climate was evaluated using the 28-item Beyond Blue School Climate Questionnaire [58]. Social support was measured using the 8-item Social Connectedness Scale [59] and the Oslo Social Support Scale [60]. Instruments that were not previously used in Nepal were translated and culturally adapted into Nepali following standardized procedures recommended for cross-cultural research [61, 62].

### Feasibility and process outcomes

The primary outcomes of this study, including feasibility, fidelity, and the implementation process, were assessed using facilitator session records, program attendance logs, and structured observations of participant engagement and implementation fidelity. Fidelity was evaluated through post-session forms completed by facilitators and supervisors, which documented the activities delivered in each session as well as any deviations from the planned content.

In addition, focus group discussions (FGDs) and in-depth interviews (IDIs) were conducted with adolescents, teachers, and facilitators to explore their perceptions and experiences regarding the feasibility, acceptability, and perceived benefits of the intervention.

### Data collection process

Four trained and experienced Research Assistants collected quantitative data by visiting selected schools, obtaining administrative approval, and distributing consent forms to students in grades 8 and 9. Eligible students returned signed caregiver consent forms and provided their assent before participating in 45–60-minute interviews conducted on Android tablets. For the qualitative aspect two experienced researchers conducted FGDs and IDIs. FGDs were facilitated by one researcher while the other took notes, and IDIs were conducted individually. FGDs were recorded and typically lasted over an hour. IDIs were conducted individually by each researcher, either on school premises or at a location convenient for participants. Participants received a notebook, pen, diary, and a token of appreciation for their participation.

### Data analysis

Quantitative data were collected using Android tablets with built-in checks to minimize missing entries and outliers, resulting in a complete dataset with no missing values. Socio-demographic characteristics such as age, sex, education, caste/ethnicity, occupation, religion, household size, and food sufficiency were summarized using descriptive statistics. As this was a feasibility trial, we only compared outcomes within each group and provided means, standard deviations, and 95% confidence intervals (CIs) for the control and intervention groups at baseline and endline. All quantitative analyses were performed using STATA. Within-group effect sizes were calculated by dividing the pre-post differences by the pooled baseline standard deviation of the outcome. Despite employing a cluster-randomized design, the analyses did not consider school-level clustering, due to the pilot nature of the feasibility trial, which was not designed to evaluate effectiveness or produce accurate cluster-adjusted estimates.

FGDs and IDIs recordings were transcribed into Nepali on-site and then translated into English by professional translators for analysis. The first author verified the transcripts for accuracy and meaning. Two researchers independently reviewed the translated transcripts to familiarize themselves with the data. Initial themes were developed based on these reviews and study objectives. Any discrepancies were resolved through discussions with the first author. Once the coding framework was established, both researchers independently coded the entire dataset using NVivo version 20. Thematic analysis was conducted, with data indexed and charted according to the thematic framework, and interpretations were made based on the charted data.

## Results

Table 1 presents the socio-demographic characteristics of participants in the pilot trial. The students’ ages ranged from 13 to 16, with a mean age of 14.6 and a standard deviation of 0.75. A slightly higher proportion of girls (52.8%) than boys participated. Most participants were from grade eight (52.8%), lived in families with 4 to 5 members (55.8%), and were Janajati (28.8%) and Brahmin/Chhetri (24.9%) caste/ethnicity. Nepali was the mother tongue for 70% of participants, and 84% were Hindus. About 9.4% of students reported working for income, and 64% were absent for 1 to 10 days in the past month. Fathers were mainly in business or services (42.5%), while mothers were predominantly in farming (64.4%). Most fathers had up to a secondary education level (44%) and nearly half of the mothers had informal education (46%). Significant differences between the control and intervention groups were observed in mother tongue, religion, father’s education level, and mother’s education level.

**Table 1:** Socio-demographic characteristics of the participants

	Control arm *N* (%)	Intervention arm *N* (%)	Total *N* (%)
*Age (years)*			
13–14	48 (42.5)	58 (48.3)	106 (45.5)
15–16	65 (57.5)	62 (51.7)	127 (54.5)
*Class/grades*			
Eight	62 (54.9)	61 (50.8)	123 (52.8)
Nine	51 (45.1)	59 (49.2)	110 (47.2)
*Gender*			
Girl	65 (57.5)	70 (58.3)	135 (57.9)
Boy	48 (42.5)	50 (41.7)	98 (42.1)
*Number of family members*			
1–3 members	7 (6.2)	12 (10)	19 (8.2)
4–5 members	62 (54.9)	68 (56.7)	130 (55.8)
More than 5	44 (38.9)	40 (33.3)	84 (36.1)
*Caste/Ethnicity*			
Brahmin/Chhetri	24 (21.2)	34 (28.3)	58 (24.9)
Janajati	19 (16.8)	48 (40.0)	67 (28.8)
Rajbanshi	48 (42.5)	4 (3.3)	52 (22.3)
Others (Dalit, Muslim, Madheshi, Majhi, Dhimal)	22 (19.5)	34 (28.3)	56 (24.0)
*Mother tongue*			
Nepali	57 (50.4)	106 (88.3)	163 (70.0)
Others (Tharu, Maithali, Rajbanshi, Bhojpuri, Dhimal, Rai, Limbu, Bangali, Magar, Newari, and others)	56 (49.6)	14 (11.7)	70 (30.0)
*Religion*			
Hindu	105 (92.9)	92 (76.7)	197 (84.5)
Others (Buddhist, Christian, Muslim, Kirat, and Atheist)	8 (7.1)	28 (23.3)	36 (15.5)
*Work for income*			
Yes	12 (10.6)	10 (8.3)	22 (9.4)
No	101 (89.4)	110 (91.7)	211 (90.6)
*Absent days in the last one month*			
None	32 (28.3)	40 (33.3)	72 (30.9)
1–10 days	73 (64.6)	77 (64.2%)	150 (64.4)
11 or more days	8 (7.1)	3 (2.5%)	11 (4.7)
*Father’s occupation*			
Farming	29 (25.7)	32 (26.7)	61 (26.2)
Work abroad	29 (25.7)	22 (18.3)	51 (21.9)
Business/ service	51 (45.1)	48 (40.0)	99 (42.5)
Unemployed	0 (0.00)	2 (1.7)	2 (0.9)
No father	4 (3.5)	16 (13.3)	20 (8.6)
*Mother’s occupation*			
Farming	73 (64.6)	77 (64.2)	150 (64.4)
Work abroad	2 (1.8)	9 (7.5)	11 (4.7)
Business/service	24 (21.2)	24 (20.0)	48 (20.6)
Unemployed	11 (9.7)	6 (5.0)	17 (7.3)
No mother	3 (2.7)	4 (3.3)	7 (3.0)
*Father’s Education*			
Illiterate	16 (14.2)	6 (5.0)	22 (9.4)
Informal education	40 (35.4)	40 (33.3)	80 (34.3)
Up to secondary level	49 (43.4)	54 (45.0)	103 (44.2)
Higher than secondary level	4 (3.5)	4 (3.3)	8 (3.4)
No father	4 (3.5)	16 (13.3)	20 (8.6)
*Mother’s Education*			
Illiterate	18 (15.9)	13 (10.8)	31 (13.3)
Informal education	56 (49.6)	52 (43.3)	108 (46.4)
Up to secondary level	36 (31.9)	45 (37.5)	81 (34.8)
Higher than secondary level	0 (0.0)	6 (5.0)	6 (2.6)
No mother	3 (2.7)	4 (3.3)	7 (3.0)
Total	113 (100.0)	120 (100.0)	233 (100.0)

**Fig. 1:**
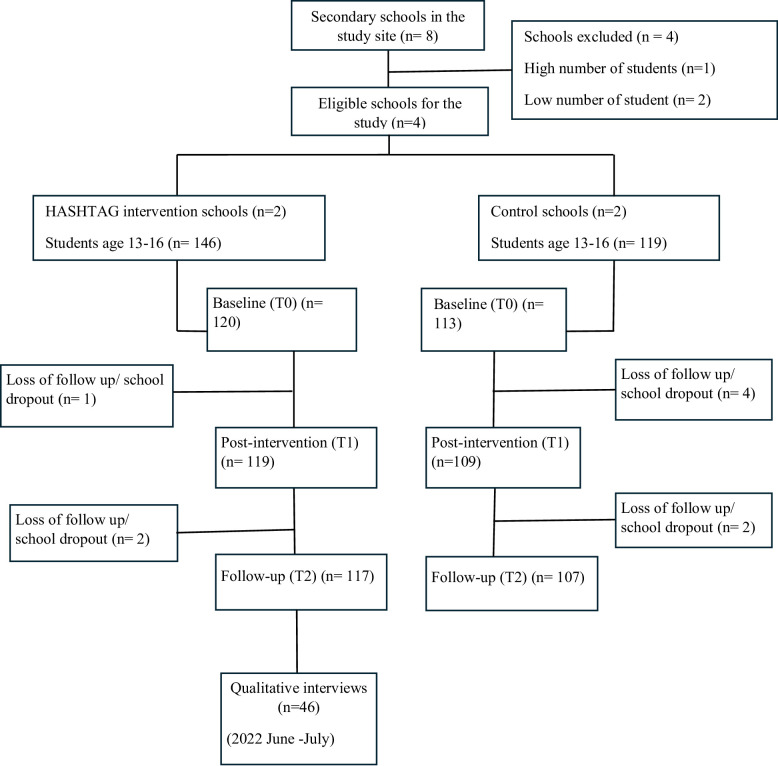
CONSORT flow chart

Figure 1 presents the consort flow diagram of the pilot trial, illustrating the count of eligible schools, students eligible for the study, and the participants in the baseline, midline, follow-up, and qualitative study.

Table 2 presents the mean, standard deviation, and 95% confidence interval, within-group effect size for baseline (T0) and post-intervention (T1) for various outcome measures for the intervention and control groups. Both groups experienced a decrease in symptoms of depression, with a slightly larger reduction in the intervention group (mean difference = -0.55) compared to the control group (mean difference = -0.35). The effect sizes (-0.13 and  -0.09) indicate small, negligible effects of the intervention. Only the intervention group showed improvement in anxiety symptoms (-0.67), with a small-to-moderate effect size (-0.21), indicating a meaningful reduction in anxiety. In contrast, the control group’s scores slightly increased at time T1 (0.28) with a very small effect size (0.09). Psychosocial functioning exhibited minimal change in the mean of both groups with effect sizes close to zero in both groups. Aggression scores decreased in the intervention group (-0.59), while they increased in the control group (0.74). The mean score on the social connectedness measure decreased in both groups, with a greater decrease in the control group (-0.59) than in the intervention group (-0.39). Conversely, social support increased in both groups, with a larger gain in the intervention group (0.36) compared to the control group (0.21). The control group reported positive changes in the school climate (1.41), while the intervention group’s score slightly decreased (-1.57). There was no meaningful difference in resilience score between the two groups. with small effect sizes (-0.14 and  -0.13). Additionally, there was a slight decrease in the well-being score in the intervention group (-0.22) and a slight increase in the control group (0.12). Bullying scores decreased slightly in the intervention group (-0.06) but increased in the control group (0.6), and the effect sizes (-0.08 and 0.07) were very small.

**Table 2:** Mean difference, and confidence intervals for different outcome measures in both intervention and control arm

Outcome measures	Intervention arm					Control arm				
	T0 mean	T1 mean	Mean difference	CI	Effect size	T0 mean	T1 mean	Mean difference	CI	Effect size
Stirling Children’s Wellbeing Scale	44.93	44.71	-0.22	-0.72–1.15	-0.03	45.06	45.17	0.12	-1.38–1.14	0.02
Adapted resilience scale	26.03	25.64	-0.39	-0.15–0.94	-0.14	26.61	26.23	-0.38	-0.20–0.96	-0.13
Patient Health Questionnaire-9 (PHQ-9)	5.11	4.56	-0.55	-0.10–1.20	-0.13	4.44	4.09	-0.35	-0.26–0.96	-0.09
Generalized Anxiety Disorder-7 (GAD-7)	4.01	3.34	-0.67	0.12–1.22	-0.21	3.04	3.32	0.28	-0.84–0.27	0.09
SDQ Prosocial behavior	8.63	8.92	0.29	-0.55 – -0.04	0.20	8.41	8.91	0.50	-0.79 – -0.20	0.35
WHODAS	8.23	8.24	0.017	-1.097–1.06	0.003	7.39	7.33	-0.055	-0.95–1.06	-0.01
AUDIT	0.30	0.22	-0.08	-0.16–0.33	-0.07	0.26	0.29	0.037	-0.26–0.19	0.03
Aggression Scale	5.28	4.69	-0.59	-0.10 − 1.28	-0.10	5.93	6.67	0.74	-2.12–0.64	0.12
Social Connectedness Scale	5.67	5.29	-0.39	-0.35–1.12	-0.09	6.30	5.71	-0.59	-0.33–1.51	-0.14
Oslo Social Support Scale	11.16	11.52	0.36	-0.71 – -0.008	0.19	11.15	11.36	0.21	-0.65–0.22	0.11
Beyond Blue School Climate Questionnaire	64.74	63.17	-1.57	-0.057–3.2	-0.14	62.20	63.61	1.41	-3.53–0.70	0.12
Gatehouse Bullying Scale	0.71	0.66	-0.06	-0.098–0.22	-0.08	0.74	0.80	0.055	-0.21–0.09	0.07

The trend of changes from pre- to post-intervention indicates that the outcome measures were responsive to change in the expected directions. Across various domains, such as depression, anxiety, aggression, social support, and bullying, the instruments captured changes consistent with anticipated improvements in the intervention group and, in some instances, showed differences from the control group. Conversely, measures like social connectedness, school climate, resilience, and well-being displayed limited or mixed changes across groups, suggesting either stability or unexpected changes over the study period. These results are interpreted as evidence of the instruments’ sensitivity to change rather than as assessment of intervention effectiveness.

### Implementation of TES

The TES program involved teacher training, School Action Groups (SAGs), and awareness raising activities to enhance the school environment and communication between students and school administration.

### Implementation of TT

#### Fidelity check

To assess the fidelity of interventions implementation, fidelity checks were conducted focusing on intervention dosage, facilitator skills, capacity, and session delivery to ensure adherence and participant engagement. A checklist based on TT content and strategies, as well as necessary session delivery skills, was used for evaluation. The field-based supervisor observed 75% of all TT sessions and held weekly meetings with facilitators for feedback and insights. Trained facilitators demonstrated a high fidelity rate of 82.2% in observed sessions. Facilitators expressed the need for more comprehensive training, especially for newcomers, suggesting the inclusion of role plays, community facilitation skills, and practical group management skills in future sessions.

#### TT session attendance

Table 3 presents the attendance of students in TT sessions, conducted in groups of 30 students comprising both boys and girls, as recommended in the formative study. On average, 70.7% of students participated in the sessions, with boys at 69% and girls at 71.9%. The highest attendance was in session 6 at 81.7% (boys 84% and girls 80%), followed by session 1 at 79.2%. The lowest attendance was in session 4 with 58.3% participation (boys 56% and girls 60%).

**Table 3:** Participation in the TT sessions by gender

Sessions	Boys (*N* = 50)	Girls (*N* = 70)	Total (*N* = 120)
Session 1	38 (76.0)	57 (81.4)	95 (79.2)
Session 2	31 (62.0)	50 (71.4)	81 (67.5)
Session 3	28 (56.0)	45 (64.3)	73 (60.8)
Session 4	28 (56.0)	42 (60.0)	70 (58.3)
Session 5	40 (80.0)	52 (74.3)	92 (76.7)
Session 6	42 (84.0)	56 (80.0)	98 (81.7)
Average	35 (69.0%)	50 (71.9%)	85 (70.7%)

**Table 4:** Three main themes encompassing eight sub-themes mapped to illustrative quotes from students, facilitators, and teachers

**Theme 1: Social and behavioral changes observed at school**
***Shifting from passive to active engagement and communication*** *Before, teachers used to tell us to clean the school during breaks, but we did not really do it. Now, we do it regularly. Personally, I have become more social and talk to almost everyone in my class, and they talk to me too. I’ve seen this change in them as well*. ***15-year-old girl***
***Improvement in student–teacher relationships*** *I have seen few changes in students. For example, students used to call out teachers’ names behind their backs, but now I haven’t heard them do so. The way they interact with teachers has also been better*. ***14-year-old girl***
***Reducing bullying and discrimination*** *Many of the students didn’t realize they were engaging in bullying behavior. They were doing these things but had not realized it was also considered bullying. We provided them with proper information about it through good content. Then they realized their actions were a form of bullying.* ***Facilitator 2***
***Identifying problems and solutions through the “Feeling Box”*** *Students used to feel shy about asking for sanitary pads provided by the school. They wrote about this in the ‘Feeling Box*,*’ and later they received them.*
**Theme 2: Key takeaways transcend the school environment**
***Learning to verbalize assertiveness*** *I liked learning to say ‘No’ because sometimes we have to refuse our friends when they ask us to do bad things. We learned how to say ‘no’.* ***14-year-old boy***
***Illuminating the importance of self-care and personal growth*** *We should love ourselves. Mostly*,* we think about what others will say about us*,* and how they will react. But self-love is important.* ***35-year-old***,*** female teacher***
**Theme 3: Challenges in implementation of HASHTAG**
***Environmental challenges*** *It was so hot in the session venue. There was no fan. It was really sunny at that time. I became sleepy.* ***14-year-old boy***
***Interpersonal dynamics among students as a challenge*** *The students who behaved badly and talked a lot during class were the ones who didn’t participate as well.* ***15-year-old boy (SAG)***

#### Learnings from TT sessions

Participants shared their feedback on various aspects of the TT intervention, including what they learned from each session. The majority reported learning skills such as standing up against pressure (*n* = 51), using 5-step problem-solving approaches (*n* = 39), respecting others (*n* = 61), measuring emotions using a thermometer (*n* = 35), expressing feelings and emotions (*n* = 20), practicing deep breathing (*n* = 27), helping others and asking for help (*n* = 11), developing communication skills (*n* = 40), engaging in active listening (*n* = 23), improving interpersonal skills [21], being assertive (*n* = 51), taking care of their own health and well-being (*n* = 29), managing stress (*n* = 55), recognizing the usefulness of stress (*n* = 14), understanding stress impact (*n* = 14), and identifying stress sources (*n* = 28).

### Qualitative results

Students and teachers found the HASHTAG intervention beneficial in various aspects of their academic and daily lives. Their perceptions were categories into social and behavioral changes at school, key takeaways beyond the school environment, and challenges in HASHTAG implementation (Table 4).

### Social and behavioral changes at school

After the TT and TES program, students, teachers and facilitators reported several social and behavioral changes at school. These included a shift from passive to more active engagement and communication, changes in student-teacher interactions, reduced bullying and discrimination, and the use of the “Feeling Box” to identify problems and solutions.

#### Shifting from passive to active engagement and communication

Both students and teachers described changes from passive to more active engagement and communication in the school setting. Teachers observed positive changes, particularly among female students who were previously hesitant to participate in class discussions, but now express themselves more confidently.

*Definitely changes have been seen especially among female students more than male students. They have realized that they should raise their voices without being scared. They have felt that this program is targeted to benefit the students and that they should be actively engaged in it.* ***50-year-old male teacher***

Students also observed positive changes in their peers, such as better discipline, greater involvement in school activities, and participation in keeping the school clean.

*I felt like this program was very nice. The school was very different when I first joined. Now it has changed a lot. I liked it because the school slowly became cleaner.* ***15-year-old boy***

Teachers similarly reported that students were more active in maintaining school cleanliness, such as using dustbins, collecting wrappers, and disposing of waste properly. Teachers also mentioned that they participated together with students in cleanliness and sanitation campaigns. A student described becoming more proactive in both communicating with classmates, and taking responsibility for cleanliness after joining SAGs.

*Before*,* teachers used to tell us to clean the school during breaks*,* but we did not really do it. Now*,* we do it regularly. Personally*,* I have become more social and talk to almost everyone in my class*,* and they talk to me too. I’ve seen this change in them as well.* ***15-year-old girl***

#### Improvement in student–teacher relationships

Students described more positive interactions and clearer communication with teachers after the program. They mentioned better understanding and feeling more comfortable approaching teachers. Students from both intervention schools shared about their experience of losing their fear of talking to teachers, especially after the establishment of the SAGs. A 14-year-old student from School A reported changes in how students spoke about teachers and interacted with them following the TT sessions.

*I have seen a few changes in students. For example*,* students used to call out teachers’ names behind their backs*,* but now I haven’t heard them do so. The way they interact with teachers has also been better.* ***14-year-old girl***

Teachers who participated in the training reflected on their earlier use of physical punishment and described changes in their approaches. Some teachers shared that they view punishment as unhelpful, and prefer discussing issues with students or involving parents or school administration when needed. A 40-year-old teacher from School B mentioned that his use of punishment had reduced to “zero or near zero” after the training. Facilitators also observed a shift in teachers’ attitudes towards punishment, with more teachers opting to engage in conversations with students to address issues rather than relying on punitive actions.

#### Reducing bullying and discrimination

Teachers, students and facilitators described reductions in bullying and discriminatory behaviours after the intervention. In both schools, participants identified name-calling and the use of offensive language as common forms of bullying. Facilitators shared that at the start of the intervention, many students did not recognize these behaviours as bullying or understand their effects. Through participation in the intervention, students developed an understanding of actions that characterize bullying.

*Many students didn’t realize they were engaging in bullying behavior. They were doing these things but had not realized it was also considered bullying. We provided them with proper information about it through good content. Then they realized their actions were a form of bullying.* ***Facilitator B***

Facilitators further noted that students became more aware of how bullying could affect others. Some students who had previously bullied others acknowledged their behaviour and expressed regret. Students from School B described how bullying at school discouraged students from attending school and negatively affected their mental health, and they felt the anti-bullying activities helped reduce these problems.

*Because of bullying*,* some students might not want to come to school. This might affect their mental health too. Conducting the anti-bullying commitment program helped in reducing this problem.* ***14-year-old boy***

After the implementation of the program, most teachers reported receiving fewer complaints related to bullying, especially after the SAGs were formed. One teacher from School B described changes in student behaviour, particularly in how boys treated girls.

*In the past*,* we used to receive several complaints about boys being disrespectful toward girls. One of the positive things I find is that SAGs were formed which has helped bring changes in students.*

The anti-bullying pledge was reported by students as meaningful, with several noting that it helped them understand the effects of bullying and the importance of respectful language. Students who had experienced bullying in the past also noticed changes, stating that classmates now spoke politely and avoided hurtful language.

#### Identifying problems and solutions through the “feeling box”

The “Feeling Box” offered students a way to anonymously share their thoughts, feelings, and daily concerns by writing them on paper and placing them in a locked box. Most participants described this as a helpful and safe way to express their problems. SAGs members explained that the Feeling Box supported communication between students and teachers by bringing forward issues students were hesitant to raise directly. Teachers shared that reading the notes helped them better understand students concerns and led to changes within the schools.

*Through the ‘Feeling Box*,*’ students were able to express what was on their minds. We were able to fix the bathroom problem and implement an anti-bullying program to address bullying.* ***40-year-old male teacher***

Facilitators also described how the Feeling Box brought attention to issues that students felt shy or uncomfortable raising openly. They reported that it was particularly useful for identifying practical needs and everyday issues.

*Students used to feel shy about asking for sanitary pads provided by the school. They wrote about this in the ‘Feeling Box*,*’ and later they received them.* ***Facilitator A***

Teachers similarly noted that while some concerns could not be addressed due to financial constraints, others were discussed with school administration and taken forward for action.

*Problems that require large financial commitments cannot be solved immediately. However*,* many issues can be addressed*,* and we have asked the administration to consider them. They are now taking steps to find solutions.* ***40-year-old male teacher***

### Key takeaways extending beyond the school environment

Both students and teachers reported key takeaways from the intervention that they were relevant beyond the school setting. Students talked about learning skills such as managing anger, showing respect towards elders, and exercising self-control. Students who participated in the TES program also mentioned learning about cleanliness, personal hygiene, and reducing the consumption of junk-food. Participants particularly emphasized two areas they felt were important: learning how to express assertiveness verbally and recognizing the importance of self-care and personal growth.

#### Learning to verbalize assertiveness

In both schools A and B, most TT participants described enjoying sessions focused on topics such as **“**saying no to friends” and “solving daily life problems.” Some TT participants from school A also mentioned sessions on “self-appraisal” and “communication skills” as particularly helpful. Students also described learning ways to express themselves clearly, especially in situations where they felt pressure from peers.

*I liked learning to say ‘No’ because sometimes we have to refuse our friends when they ask us to do bad things. We learned how to say ‘no’.****14-year-old boy***

#### Illuminating the importance of self-care and personal growth

Participants reported making changes in their daily life activities following the intervention. Several students and teachers reported paying more attention to politeness, discipline, problem-solving, and communicating confidently in their everyday lives. One teacher described learning about ‘self-love’ as the most meaningful part of the intervention, and shared that it had become an important part of her personal life.

*We should love ourselves. Mostly*,* we think about what others will say about us*,* and how they will react. But self-love is important.* ***35-year-old***,*** female teacher***

Along with self-love, participants also spoke about other personal values and character-oriented priorities that they felt had become more important after taking part in the intervention.

### Challenges in the implementation of HASHTAG

Although students, teachers and facilitators described several benefits of the HASHTAG intervention, they also reported multiple challenges during its implementation. These challenges were mainly related to environmental conditions and interpersonal dynamics among students.

#### Environmental challenges

Students frequently reported challenges related to the physical environment in which sessions were conducted. Many reported feeling uncomfortable due to poor ventilation, high temperatures, long sessions without breaks, and limited seating arrangements. Some students also noted that the space was sometimes too small and crowded, making it difficult to sit comfortably or maintain distance.

*It was so hot in the session venue. There was no fan. It was really sunny at that time. I became sleepy.* ***14-year-old boy***

Facilitators shared similar concerns, explaining that students often had to sit on the floor, which was particularly uncomfortable for female students wearing skirts. Facilitators also reported that sessions were frequently disrupted by loudspeaker announcement related to the election campaign, which made it difficult for students to concentrate. Teachers emphasized that adequate space, ventilation, and seating are important for delivering sessions effectively.

*Students couldn’t receive all the information we wanted to provide because of loudspeaker advertisements on the road. This was the major disturbance.* ***Facilitator C***

Teachers also described challenges related to limited resources, including a lack of sufficient food and drinking water during sessions, which affected the smooth running of activities.

*Sometimes while conducting TT sessions*,* there was not enough water to drink*,* which disturbed the sessions.* ***53-year-old male teacher***

The COVID-19 pandemic further affected implementation, as some participants were absent from school due to closures or concern about infection. Students also described discomfort related to wearing face masks during sessions, especially because of sweating.

*We had to wear masks all the time*,* and we were sweaty inside.* ***14-year-old boy***

#### Interpersonal dynamics among students as a challenge

Students also described challenges related to interpersonal dynamics. One concern raised was linked to the “Feeling Box”, where some students felt their anonymity was compromised. A student reported that a member of SAG shared information from a “Feeling Box” and identified the writer based on handwriting.

*SAGs members told others about the issues written in the Feeling Box and shared them with friends. They figured out who wrote the note by recognizing the handwriting.* ***14-year-old girl***

Some students avoided sessions due to their length or because sessions were scheduled on holidays or Saturdays. In some cases, students reported skipping sessions by staying in restrooms. Facilitators also reported low attendance during holidays and fairs, which made it difficult to deliver session as planned. Teachers echoed these concerns and suggested that the sessions should not be scheduled during holidays, as students generally expect these times to use for rest and leisure (Table 4).

## Discussion

This study evaluated the feasibility and acceptability of implementing the HASHTAG school-based mental health promotion intervention in four secondary schools in eastern Nepal. Overall, the findings indicate that a program combining whole-school strategies (TES) with targeted group sessions (TT) can be delivered in schools by trained facilitators, with strong engagement from students and staff, despite substantial disruption due to COVID-19. Such multi-component designs align with the public health approach recommended for LMIC school settings, where promotion and prevention strategies can be embedded within education systems to reach a large number of adolescents [63]. The average attendance for the TT was 71%, with girls attending at a rate of 72% and boys at 69%. Absence was primarily due to disruptions caused by the pandemic and other competing responsibilities, which are common challenges for sustained participation in school-based programs in low-resource settings [63, 64]. Recruitment and retention rates were high, with minimal loss to follow-up, mainly due to school dropout, indicating strong acceptance of the intervention and study procedure among adolescents and schools. Students reported gaining a range of skills including coping with pressure, problem-solving, empathy, emotional regulation, deep breathing, peer support, communication, active listening, assertiveness, stress management, and stress awareness. Descriptive analyses indicated promising trends, indicating reductions in symptoms of depression, anxiety, aggression, and bullying. However, changes across groups were limited or mixed, suggesting stability or unexpected variations over the study period.

This study in Nepal is the first to explore both whole school climate and targeted mental health promotion interventions. Similar to findings from LMICs and HICs, participants in our study stressed the importance of addressing both the broader school environment and individual-level skills through an integrated approach [35, 64, 65]. They highlighted the equal importance of both the TES and TT components. TES was crucial in improving the overall school climate and enhancing staff-student relationships, which align with previous research evidence linking school climate improvements to better student well-being and social outcomes [65]. On the other hand, the TT intervention was seen as particularly effective in promoting social and behavioural change and enhancing learning behaviours, consistent with evidence supporting skill-based interventions for adolescents [66]. The training of facilitators was found to well-received by both students and teachers, indicating its feasibility in settings with limited mental health resources [23, 67]. This finding aligns with growing body of evidence from LMICs demonstrating that adequately trained non-specialist providers can effectively deliver psychosocial interventions and are generally well-received by participants [66, 68, 69].

A key implementation challenge was the lack of suitable physical facilities for the TT sessions. Many schools did not have dedicated spaces with proper ventilation, cooling, and seating arrangements. Some sessions had to be held on floor mats, which presented practical challenges, especially for girls due to school dress codes and limited opportunities for interactive learning. Interestingly, participants did not report mental health stigma as a barrier to implementation, which contrasts with previous research [28, 70]. This could be attributed to a culturally sensitive adaptation process [38], using non-stigmatizing languages such as *“heart-mind”* and *“distress”* [71]. The program’s name *“Sangai Phulau*,* Phalaum”* and the consistent use of inclusive terminology by teachers, facilitators, SAGs members likely created a supportive non-judgmental environment, reducing the risk labelling and stigma. These findings align with previous studies showing that adapting interventions and using non-stigmatized language can improve acceptability and effectiveness among children and adolescents in various settings [64, 72, 73].

Participants provided several practical recommendations to improve acceptability and scalability. These included integrating breathing exercises into school assemblies, avoiding sessions during breaks or holidays, aligning the intervention with the academic calendar (starting in April/Baisakh), shortening session durations and increasing interactivity. Improvements to physical infrastructure such as ensuring adequate space, ventilation, and seating, were also emphasized. Participants further recommended expanding content to address contextual challenges affecting adolescents in Nepal, including child marriage, gender-based violence, gender inequalities and adolescent development changes [74]. Additional suggestion included strengthening teacher training and incorporating more stress management content. These adaptations could enhance relevance and scalability, particularly in alignment with the National Mental Health Strategy and Action Plan 2020. This plan aims to integrate mental health education into the school curriculum in partnership with the Ministry of Education [75], and the National Adolescent Health and Development Strategy [33].

The study has several limitations. First, the TES component of the intervention was implemented for a shorter duration than initially planned due to COVID-19 restriction, limiting the assessment of its feasibility over a longer period. Second, the intervention was conducted in only two public schools with smaller class sizes, which may affect the generalizability of the findings to schools with larger student populations or private schools with different educational settings. Third, the small sample size prevented statistical analyses to evaluate changes in symptom severity, mental well-being, and other outcomes between the intervention and control groups. Fourth, while a cluster-randomized design was used in this pilot study, clustering at the school level was not taken into account in the analysis. The study was not intended to measure effectiveness or provide precise cluster-adjusted estimates, so the quantitative results should be viewed as exploratory. Fifth, although the qualitative interviews were conducted by trained researchers not involved in other study components, participants may have provided overly positive responses due to social desirability bias. Similarly, the quantitative results may have been impacted by short follow up duration and participant self-report bias. Finally, the pilot study showed promising results, a fully powered randomized controlled trial across multiple districts, with a longer follow-up period is needed to robustly evaluate the intervention’s long-term effect.

## Conclusion

This feasibility study suggests that the HASHTAG program is a promising and acceptable school-based mental health promotion program for adolescents aged 13 to 16 years. The findings indicate that the intervention can be delivered by non-specialists and is associated with high engagement and perceived benefits in social and behavioural skills. Key areas for improvement include strengthening logistic arrangements, improving physical infrastructure, incorporating parental involvement, extending implementation across the academic year, aligning delivery with school calendars, and optimizing session duration and interactivity. Addressing these factors will be important for enhancing feasibility and scalability. While preliminary findings suggest potential benefits, a fully powered randomized controlled trial (RCT) is required to establish the effectiveness of the intervention and assess its longer-term impact on adolescents’ mental health and well-being.

## Data Availability

Interested individuals can contact the investigator of this study to express their interest in collaboration and request access to the dataset analysed here by emailing: [luitelnp@gmail.com](mailto: luitelnp@gmail.com).

## Abbreviations

COVID-19: COronaVIrus Disease of 2019
FGD: Focus Group Discussion
HASHTAG: Health Action in Schools for aThriving Adolescent Generation
IDI: Individual Interview
LMIC: Low- and Middle-Income Countries
RCT: Randomized Controlled Trial
SAG: School Action Group
TES: Thriving Environment in School
TT: Thriving Together

## References

[1] UNICEF. UNICEF Annual Report 2024. New York: United Nations Children’s Fund; 2025.

[2] Shinde S, Harling G, Assefa N, Bärnighausen T, Bukenya J, Chukwu A, et al. Counting adolescents in: the development of an adolescent health indicator framework for population-based settings. EClinicalMedicine. 2023;61:102067. 37448809 10.1016/j.eclinm.2023.102067 PMC10336247

[3] WHO. Mental health of adolescents. 2025. https://www.who.int/news-room/fact-sheets/detail/adolescent-mental-health.

[4] Kessler RC, Amminger GP, Aguilar-Gaxiola S, Alonso J, Lee S, Ustün TB. Age of onset of mental disorders: a review of recent literature. Curr Opin Psychiatry. 2007;20(4):359–64. 17551351 10.1097/YCO.0b013e32816ebc8c PMC1925038

[5] Kieling C, Buchweitz C, Caye A, Silvani J, Ameis SH, Brunoni AR, et al. Worldwide Prevalence and Disability From Mental Disorders Across Childhood and Adolescence: Evidence From the Global Burden of Disease Study. JAMA psychiatry. 2024;81(4):347–56. 38294785 10.1001/jamapsychiatry.2023.5051 PMC10831630

[6] Thapar A, Eyre O, Patel V, Brent D. Depression in young people. Lancet (London England). 2022;400(10352):617–31. 35940184 10.1016/S0140-6736(22)01012-1

[7] Erskine HE, Baxter AJ, Patton G, Moffitt TE, Patel V, Whiteford HA, et al. The global coverage of prevalence data for mental disorders in children and adolescents. Epidemiol Psychiatr Sci. 2017;26(4):395–402. 26786507 10.1017/S2045796015001158 PMC6998634

[8] Lund C, Abrahams Z, Garman E, van de Westhuizen C, Sorsdahl K. Environment matters: The social determinants of child and adolescent mental health. Cape Town, South Africa: Children’s Institute, University of Cape Town; 2021.

[9] Fatori D, Bordin IA, Curto BM, de Paula CS. Influence of psychosocial risk factors on the trajectory of mental health problems from childhood to adolescence: a longitudinal study. BMC Psychiatry. 2013;13:31. 23327711 10.1186/1471-244X-13-31 PMC3570478

[10] Luitel NP, Jordans MJD, Adhikari A, Upadhaya N, Hanlon C, Lund C, et al. Mental health care in Nepal: current situation and challenges for development of a district mental health care plan. Confl health. 2015;9:3. 25694792 10.1186/s13031-014-0030-5 PMC4331482

[11] Dhimal M, Dahal S, Adhikari K, Koirala P, Bista B, Luitel N, et al. A Nationwide Prevalence of Common Mental Disorders and Suicidality in Nepal: Evidence from National Mental Health Survey, 2019–2020. J Nepal Health Res Counc. 2022;19(4):740–7. 35615831 10.33314/jnhrc.v19i04.4017

[12] Sharma V, Levin BL, Rahill GJ, Baldwin JA, Luitel A, Marhefka SL. Post-earthquake Self-Reported Depressive Symptoms and Post-Traumatic Stress Disorder and their Correlates among College-Youths in Kathmandu, Nepal. Psychiatr Q. 2021;92(4):1595–609. 34109493 10.1007/s11126-021-09928-5 PMC8189706

[13] Risal A, Sharma PP. Psychiatric illness in the paediatric population presenting to a psychiatry clinic in a tertiary care centre. Kathmandu Univ Med J. 2010;8(32):375–3981. 10.3126/kumj.v8i4.6235 22610765

[14] Ojha SP, Ma J, Chapagain M, Tulachan P. Emotional and behavioural problems among sheltered homeless children. JNMA. 2013;52(191):457–61. 24907950

[15] Silwal S, Dybdahl R, Chudal R, Sourander A, Lien L. Psychiatric symptoms experienced by adolescents in Nepal following the 2015 earthquakes. J Affect Disord. 2018;234:239–46. 29549825 10.1016/j.jad.2018.03.002

[16] Gautam P, Dahal M, Ghimire H, Chapagain S, Baral K, Acharya R, et al. Depression among Adolescents of Rural Nepal: A Community-Based Study. Depress Res Treat. 2021;2021:7495141. 33628501 10.1155/2021/7495141 PMC7880710

[17] Karki A, Thapa B, Pradhan PMS, Basel P. Depression, anxiety and stress among high school students: A cross-sectional study in an urban municipality of Kathmandu, Nepal. PLOS global public health. 2022;2(5):e0000516. 36962418 10.1371/journal.pgph.0000516 PMC10022099

[18] Paudel S, Gautam H, Adhikari C, Yadav DK. Depression, Anxiety and Stress among the Undergraduate Students of Pokhara Metropolitan, Nepal. J Nepal Health Res Counc. 2020;18(1):27–34. 32335589 10.33314/jnhrc.v18i1.2189

[19] Rimal H, Pokharel A. Prevalence of Attention Deficit Hyperactivity Disorder among School Children and Associated Co-morbidities - A Hospital Based Descriptive Study. Kathmandu Univ Med J. 2016;14(55):226–30. 28814683

[20] Dangal MR, Bajracharya LS. Students Anxiety Experiences during COVID-19 in Nepal. Kathmandu Univ Med J. 2020;18(70):53–7. 33605239

[21] Schäfer SK, Streit S, Schäfer CG, Roembell LB, Corneli M, Schaubruch LM, et al. Barriers and facilitators for the implementation of preventative mental health interventions among secondary schools in high-income countries: a systematic review. Eur Child Adolesc Psychiatry. 2025;34(12):3713–31. 40586958 10.1007/s00787-025-02796-5 PMC12743069

[22] Kirnan J, Fotinos G, Pitt K, Lloyd G. School-based mental health education: program effectiveness and trends in help-seeking. Int J Environ Res Public Health. 2025;22(4):523. 40283749 10.3390/ijerph22040523 PMC12027138

[23] Luitel NP, Jordans MJ, Adhikari A, Upadhaya N, Hanlon C, Lund C, et al. Mental health care in Nepal: current situation and challenges for development of a district mental health care plan. Confl health. 2015;9:3. 25694792 10.1186/s13031-014-0030-5 PMC4331482

[24] Luitel NP, Jordans MJD, Kohrt BA, Rathod SD, Komproe IH. Treatment gap and barriers for mental health care: A cross-sectional community survey in Nepal. PLoS ONE. 2017;12(8):e0183223. 28817734 10.1371/journal.pone.0183223 PMC5560728

[25] Khanal G, Selvamani Y, Sapkota P. Exploring barriers and facilitators of mental health care in Sudurpaschim Province, Nepal: a socioecological qualitative study of patients with depression and anxiety and health care professionals. BMC Health Serv Res. 2025;25(1):855. 40597294 10.1186/s12913-025-12983-4 PMC12219968

[26] Shrestha A, Poudel DB, Thapa P. The Impact of Sambhavya-SEL: Strengthening Social and Emotional Competence in Middle School Students 680–699. Creative Educ. 2025;16(5):680–99.

[27] Khanal SP, Budhathoki CB, Okan O. Effectiveness of a school-based health literacy intervention in improving adolescent health literacy and the intention to take health-promoting actions. BMC Public Health. 2025;25(1):3551. 41121137 10.1186/s12889-025-24827-1 PMC12542496

[28] Rose-Clarke K, KP B, Magar J, Pradhan I, Shrestha P, Hassan E, et al. School-based group interpersonal therapy for adolescents with depression in rural Nepal: a mixed methods study exploring feasibility, acceptability, and cost. Global mental health (Cambridge England). 2022;9:416–28. 36618751 10.1017/gmh.2022.46 PMC9806967

[29] Pacini A, Broker H, Shrestha P. Lost in translation? Cultural adaptation of child mental health interventions in Nepal: A systematic review. J Child Fam stud. 2024;33:2705–16.

[30] McDaid D, Park AL, Wahlbeck K. The Economic Case for the Prevention of Mental Illness. Annu Rev Public Health. 2019;40:373–89. 30601725 10.1146/annurev-publhealth-040617-013629

[31] Beames JR, Kikas K, Werner-Seidler A. Prevention and early intervention of depression in young people: an integrated narrative review of affective awareness and Ecological Momentary Assessment. BMC Psychol. 2021;9(1):113. 34392830 10.1186/s40359-021-00614-6 PMC8365890

[32] Barry MM, Clarke AM, Jenkins R, Patel V. A systematic review of the effectiveness of mental health promotion interventions for young people in low and middle income countries. BMC Public Health. 2013;13:835. 24025155 10.1186/1471-2458-13-835 PMC3848687

[33] MOHP. National Adolescent Health and Development Strategy Kathmandu. Ministry of Health and Population 2019.

[34] Laurenzi CA, du Toit S, Mawoyo T, Luitel NP, Jordans MJD, Pradhan I, et al. Development of a school-based programme for mental health promotion and prevention among adolescents in Nepal and South Africa. SSM Mental health. 2024;5:100289. 38910844 10.1016/j.ssmmh.2023.100289 PMC11188151

[35] Margaretha M, Azzopardi PS, Fisher J, Sawyer SM. School-based mental health promotion: A global policy review. Front Psychiatry. 2023;14:1126767. 37139309 10.3389/fpsyt.2023.1126767 PMC10149729

[36] Hopewell S, Chan AW, Collins GS, Hróbjartsson A, Moher D, Schulz KF, et al. CONSORT 2025 statement: updated guideline for reporting randomised trials. BMJ (Clinical Res ed). 2025;389:e081123. 10.1136/bmj-2024-081123 PMC11995449 40228833

[37] National Statistics Office. National Population and Housing Census 2021. Kathmandu: Government of Nepal, Office of the Prime Minister and Council of Ministers. 2023. https://censusnepal.cbs.gov.np/results/literacy. Accessed 30 Apr 2023.

[38] Sangraula M, Kohrt BA, Ghimire R, Shrestha P, Luitel NP, van’t Hof E, et al. Development of the mental health cultural adaptation and contextualization for implementation (mhCACI) procedure: a systematic framework to prepare evidence-based psychological interventions for scaling. Global Ment Health. 2021;8:e6. 10.1017/gmh.2021.5 PMC8082944 33996110

[39] Bernal G, Bonilla J, Bellido C. Ecological validity and cultural sensitivity for outcome research: issues for the cultural adaptation and development of psychosocial treatments with Hispanics. J Abnorm Child Psychol. 1995;23(1):67–82. 7759675 10.1007/BF01447045

[40] Skeen S, Laurenzi CA, Gordon SL, du Toit S, Tomlinson M, Dua T, et al. Adolescent mental health program components and behavior risk reduction: a meta-analysis. Pediatrics. 2019;144(2):e20183488. 31262779 10.1542/peds.2018-3488

[41] Sharma S, Mahotra A, Thapa TR, Thapa P, Bhandary S, Bhushal S, et al. Factors influencing utilization of school health nurse program among secondary students of Lalitpur, Nepal: a mixed-method study. BMC Public Health. 2025;25(1):771. 40001038 10.1186/s12889-025-21972-5 PMC11853607

[42] Pedersen GA, Shrestha P, Akellot J, Sepulveda A, Luitel NP, Kasujja R, et al. A mixed methods evaluation of a World Health Organization competency-based training package for foundational helping skills among pre-service and in-service health workers in Nepal, Peru and Uganda. Global Ment Health. 2023;10:e55. 10.1017/gmh.2023.43 PMC10579647 37854401

[43] Singh R, Chhetri K, Khanal P, Maharjan S, Garman E, Jordans MJD, et al. Assessment and management of suicidality in a mental health survey among poverty affected adolescents in Nepal. BMC Public Health. 2025;25(1):3612. 41146168 10.1186/s12889-025-24943-y PMC12560324

[44] Liddle I, Carter GFA. Emotional and psychological well-being in children: The development and validation of the Stirling Children’s Well-being Scale. Educational Psychol Pract. 2015;31(2):174–85.

[45] Johnson JG, Harris ES, Spitzer RL, Williams JB. The patient health questionnaire for adolescents: validation of an instrument for the assessment of mental disorders among adolescent primary care patients. J Adolesc health: official publication Soc Adolesc Med. 2002;30(3):196–204. 10.1016/s1054-139x(01)00333-0 11869927

[46] Spitzer RL, Kroenke K, Williams JB, Löwe B. A brief measure for assessing generalized anxiety disorder: the GAD-7. Arch Intern Med. 2006;166(10):1092–7. 16717171 10.1001/archinte.166.10.1092

[47] Luitel NP, Rimal D, Eleftheriou G, Rose-Clarke K, Nayaju S, Gautam K, et al. Translation, cultural adaptation and validation of Patient Health Questionnaire and generalized anxiety disorder among adolescents in Nepal. Child Adolesc Psychiatry Mental Health. 2024;18(1):74. 10.1186/s13034-024-00763-7 PMC11188246 38898474

[48] Goodman R. The strengths and difficulties questionnaire. 1997. https://www.sdqinfo.org/py/sdqinfo/b3.py?language=Nepali.

[49] Jordans MJD, Komproe IH, Tol WA, Kohrt BA, Luitel NP, Macy RD, et al. Evaluation of a classroom-based psychosocial intervention in conflict-affected Nepal: a cluster randomized controlled trial. J Child Psychol Psychiatry. 2010;51(7):818. 20102428 10.1111/j.1469-7610.2010.02209.x

[50] Schmidt A, Barry KL, Fleming MF. Detection of problem drinkers: the Alcohol Use Disorders Identification Test (AUDIT). South Med J. 1995;88(1):52–9. 7817228

[51] Pradhan B, Chappuis F, Baral D, Karki P, Rijal S, Hadengue A, et al. The alcohol use disorders identification test (AUDIT): validation of a Nepali version for the detection of alcohol use disorders and hazardous drinking in medical settings. Subst Abuse Treat Prev Policy. 2012;7:42. 23039711 10.1186/1747-597X-7-42 PMC3508982

[52] Luitel NP, Baron EC, Kohrt BA, Komproe IH, Jordans MJD. Prevalence and correlates of depression and alcohol use disorder among adults attending primary health care services in Nepal: a cross sectional study. BMC Health Serv Res. 2018;18(1):215. 29587724 10.1186/s12913-018-3034-9 PMC5869789

[53] WHO. WHO Disability Assessment Schedule 2.0: children and youth 36–item version. https://medicine.usask.ca/documents/psychiatry/WHODAS2_20150123-1.pdf2010.

[54] Orpinas P, Frankowski R. The Aggression Scale: A self-report measure of aggressive behavior for young adolescents. J Early Adolescence. 2001;21(1):50–67.

[55] Wagnild GM, Young HM. Development and psychometric evaluation of the Resilience Scale. J Nurs Meas. 1993;1(2):165–78. 7850498

[56] Kohrt BA, Worthman CM, Adhikari RP, Luitel NP, Arevalo JM, Ma J, et al. Psychological resilience and the gene regulatory impact of posttraumatic stress in Nepali child soldiers. Proc Natl Acad Sci U S A. 2016;113(29):8156–61. 27402736 10.1073/pnas.1601301113 PMC4961140

[57] Bond L, Wolfe S, Tollit M, Butler H, Patton G. A comparison of the Gatehouse Bullying Scale and the peer relations questionnaire for students in secondary school. J Sch Health. 2007;77(2):75–9. 17222158 10.1111/j.1746-1561.2007.00170.x

[58] Sawyer MG, Pfeiffer S, Spence SH, Bond L, Graetz B, Kay D, et al. School-based prevention of depression: a randomised controlled study of the beyondblue schools research initiative. J Child Psychol Psychiatry Allied Discip. 2010;51(2):199–209. 10.1111/j.1469-7610.2009.02136.x 19702662

[59] Lee RM, Robbins SB. The Social Connectedness and the Social Assurance scales. J Couns Psychol. 1995;42(2):232–41.

[60] Kocalevent RD, Berg L, Beutel ME, Hinz A, Zenger M, Härter M, et al. Social support in the general population: standardization of the Oslo social support scale (OSSS-3). BMC Psychol. 2018;6(1):31. 30016997 10.1186/s40359-018-0249-9 PMC6050647

[61] van Ommeren M, Sharma B, Thapa SB, Makaju R, Prasain D, Bhattarai R, et al. Preparing Instruments for Transcultural Research: Use of the Translation Monitoring Form with Nepali-Speaking Bhutanese Refugees. Transcult Psychiatry. 1999;36(3):285–301.

[62] Kohrt BA, Jordans MJ, Tol WA, Luitel NP, Maharjan SM, Upadhaya N. Validation of cross-cultural child mental health and psychosocial research instruments: adapting the Depression Self-Rating Scale and Child PTSD Symptom Scale in Nepal. BMC Psychiatry. 2011;11(1):127. 21816045 10.1186/1471-244X-11-127 PMC3162495

[63] Fazel M, Patel V, Thomas S, Tol W. Mental health interventions in schools in low-income and middle-income countries. lancet Psychiatry. 2014;1(5):388–98. 26361001 10.1016/S2215-0366(14)70357-8

[64] Harte P, Barry MM. A scoping review of the implementation and cultural adaptation of school-based mental health promotion and prevention interventions in low-and middle-income countries. Global Ment Health. 2024;11:e55. 10.1017/gmh.2024.48 PMC11094552 38751723

[65] Moore G. Annual Research Review: Improving school climate to improve child and adolescent mental health and reduce inequalities. J Child Psychol Psychiatry Allied Discip. 2026;67(4):566–87. 10.1111/jcpp.70061 PMC13036393 41077548

[66] Grande AJ, Hoffmann MS, Evans-Lacko S, Ziebold C, de Miranda CT, McDaid D, et al. Efficacy of school-based interventions for mental health problems in children and adolescents in low and middle-income countries: A systematic review and meta-analysis. Front Psychiatry. 2022;13:1012257. 36684024 10.3389/fpsyt.2022.1012257 PMC9852982

[67] Rai Y, Gurung D, Gautam K. Insight and challenges: mental health services in Nepal. BJPsych Int. 2021;18(2):E5. 34287402 10.1192/bji.2020.58 PMC8274424

[68] Rose AL, Jack HE, Wan C, Toloza E, Bhattiprolu K, Ragunathan M, et al. Implementing task-shared child and adolescent psychological interventions in low- and middle-income countries: a scoping review. J Clin Child Adolesc Psychol. 2025;54(1):83–98. 36507739 10.1080/15374416.2022.2151450 PMC10258230

[69] Terp AM, Habashneh R, Brown FL, Abualhaija A, Aqel IS, Ghatasheh M, et al. Facilitators and barriers to participation and scale-up of a non-specialist delivered psychological intervention for adolescents in low-resourced settings: a process evaluation. BMC Public Health. 2025;25(1):725. 39984951 10.1186/s12889-025-21914-1 PMC11846470

[70] Girio-Herrera E, Ehrlich CJ, Danzi BA, La Greca AM. Lessons Learned About Barriers to Implementing School-Based Interventions for Adolescents: Ideas for Enhancing Future Research and Clinical Projects. Cogn Behav Pract. 2019;26(3):466–77. 32855590 10.1016/j.cbpra.2018.11.004 PMC7448397

[71] Kohrt BA, Hruschka DJ. Nepali concepts of psychological trauma: the role of idioms of distress, ethnopsychology and ethnophysiology in alleviating suffering and preventing stigma. Cult Med Psychiatry. 2010;34(2):322–52. 20309724 10.1007/s11013-010-9170-2 PMC3819627

[72] Mishu MP, Tindall L, Kerrigan P, Gega L. Cross-culturally adapted psychological interventions for the treatment of depression and/or anxiety among young people: A scoping review. PLoS ONE. 2023;18(10):e0290653. 37878658 10.1371/journal.pone.0290653 PMC10599551

[73] Hartog K, Peters RMH, Tukahiirwa RK, Jordans MJD. Reducing stigma impacting children and adolescents in low- and middle-income countries: The development of a common multi-component stigma reduction intervention. PLoS ONE. 2023;18(10):e0292064. 37906579 10.1371/journal.pone.0292064 PMC10617710

[74] UNICEF. Nepal: 2024 annual results report – UNFPA–UNICEF global programme to end child marriage. https://www.unicef.org/media/172906/file/Nepal.pdf.pdf2024.

[75] MoHP. National Mental Health Strategy and Action Plan 2020. Kathmandu, Nepal: Government of Nepal Ministry of Health and Population; 2020.

